# In Vitro Anti-Inflammatory and Skin Protective Effects of *Codium fragile* Extract on Macrophages and Human Keratinocytes in Atopic Dermatitis

**DOI:** 10.4014/jmb.2312.12002

**Published:** 2024-01-17

**Authors:** A-yeong Jang, JeongUn Choi, Weerawan Rod-in, Ki Young Choi, Dae-Hee Lee, Woo Jung Park

**Affiliations:** 1 Department of Marine Bio Food Science, Gangneung-Wonju National University, Gangneung 25457, Republic of Korea; 2 Department of Agricultural Science, Faculty of Agriculture Natural Resources and Environment, Naresuan University, Phitsanulok 65000 Thailand; 3 Nbios Inc., Gangneung, Gangwon 25457, Republic of Korea

**Keywords:** *Codium fragile*, barrier function, inflammation, atopic dermatitis

## Abstract

*Codium fragile* has been traditionally used in oriental medicine to treat enterobiasis, dropsy, and dysuria, and it has been shown to possess many biological properties. Atopic dermatitis (AD) is one of the types of skin inflammation and barrier disruption, which leads to chronic inflammatory skin diseases. In the current investigation, the protective effects of *C. fragile* extract (CFE) on anti-inflammation and skin barrier improvement were investigated. In LPS-stimulated RAW 264.7 cells, nitric oxide generation and the expression levels of interleukin *(IL)-1β*, *IL-4*, *IL-6*, *iNOS*, *COX-2*, and tumor necrosis factor-alpha *(TNF)-α* were reduced by CFE. CFE also inhibited the phosphorylation of NF-κB-p65, ERK, p-38, and JNK. Additionally, CFE showed inhibitory activity on *TSLP* and *IL-4* expression in HaCaT cells stimulated with TNF-α/interferon-gamma (IFN-γ). Enhanced expression of factors related to skin barrier function, FLG, IVL, and LOR, was confirmed. These findings implied that CFE may be used as a therapeutic agent against AD due to its skin barrier-strengthening and anti-inflammatory activities, which are derived from natural marine products.

## Introduction

Atopic dermatitis (AD) is a recurrent or chronic inflammatory skin disease, which has been related to sleep issues, emotional instability, eczematous lesions, itching, erythema, and dry skin [1, 2]. Infants and young children with AD exhibit symptoms, which trigger an atopic march that progresses to other allergy disorders, such asthma and allergic rhinitis [3]. Certain common symptoms of AD include redness, itching, thickening of the skin, and dryness of the skin due to inflammatory reactions [1]. It is mediated by migration of cells associated with inflammation, such as macrophages, eosinophils, and mast cells [4]. Skin consists of the following four distinct layers: the epidermis, subcutaneous tissue, dermis, and muscle. Particularly, the epidermis is the outer layer of the skin and it serves as the primary barrier against external stimuli. Keratinocytes constitute 95% of the epidermis and produce factors related to skin barrier as well as they play a critical part in inflammation and immunological responses [5]. Molecules related to skin barrier, such as filaggrin (FLG), involucrin (IVL), and loricrin (LOR), regulate skin permeability and prevent moisture evaporation, which prevents AD and various inflammatory skin disorders [6]. Many studies have demonstrated that FLG, IVL, and LOR expression is reduced in atopic skin [6-8]. In patients with AD, the innate and acquired immune systems react abnormally, resulting in an allergic inflammatory reaction [9].

Inflammation is a host defense reaction triggered by physical activity, chemicals, bacterial infection, or pathogen invasion [10]. In chronic inflammatory states, the body continues to respond to inflammation, leading to illnesses, such as diabetes, cardiovascular disease, arthritic conditions, and AD [11]. Macrophages are innate immune cells that play a significant role in inflammation by causing synthesis of inflammatory mediators, such as cyclooxygenase-2 (COX-2) and inducible nitric oxide synthase (iNOS), as well as by regulating the production of pro-inflammatory cytokines, such as interleukin (IL)-6, tumor necrosis factor-alpha (TNF-α), and IL-1β [12]. The expression of these inflammatory mediators is promoted by nuclear factor kappa B (NF-κB) and mitogen-activated protein kinases (MAPK), which mediate the crucial cellular mechanism involved in anti-inflammatory actions [13]. Numerous investigations have found that a variety of substances exert anti-inflammatory effects by preventing the NF-κB and MAPK cascades [14-16].

*Codium fragile* is an edible seaweed from the Codiaceae family, which is found throughout the coasts of East Asia, Oceania, and North Europe [17]. *C. fragile* has been used to treat enterobiasis, dropsy, and dysuria, and it possesses anti-coagulant [18], anti-inflammatory [19], immunostimulatory [17, 20], anti-cancer [21], anti-obesity [22], and antiviral activities [23]. In a recent study, a methanol extract of *C. fragile* suppressed lipopolysaccharide (LPS)-induced secretion of nitric oxide (NO), prostaglandin E2 (PGE_2_), iNOS, COX-2, and TNF-α via NF-κB activation in RAW 264.7 cells, leading to anti-inflammatory effects [24].

However, the anti-inflammatory effect of *C. fragile* extract (CFE) via regulation of skin protection against AD is still unknown. Numerous investigations have proved that natural bioactive substances derived from marine algae can be used to improve the epidermal skin physiology in the therapy of AD [25]. Consequently, the purpose of this study was to evaluate the AD improvement effect of CFE on cellular mechanisms and skin barrier reactivity related to anti-inflammatory properties in LPS-stimulated RAW 264.7 cells and TNF-α/IFN-γ stimulated HaCaT cells.

## Materials and Methods

### Preparation of CFE

Green algae (*C. fragile*) in freeze-dried powder form was purchased and used from the Biocorp Co.,Ltd.(Republic of Korea). The dried material (300 g) was crushed and extracted with 4 L of 50% EtOH at 80 rpm for 24 h. The solution was filtered, evaporated, and freeze-dried (24 g, yield 8%). The extraction process of the ethyl acetate fraction was as described (Fig. S1). The chromatographic peaks at No. 5 were chosen for the subsequent experiment, which produced CFE, after the purity of the EA fractions of CFE was evaluated by high-performance liquid chromatography (HPLC) analysis. The dried extract of CEF was kept at 4°C until utilized.

### Identification Analysis of Guanosine and Uridine in CFE

To identify and quantify guanosine and uridine obtained from CFE, HPLC analysis was performed to analyze CFE compared with their standards at concentrations of 30, 100, 500, and 1000 μg/ml. Before injecting into the HPLC system, CFE (15 mg) diluted in 1 ml of methanol and standard solutions were filtered using 0.22 μm syringe filters. CFE (20 μl) was injected and analyzed using a Shimadzu Nexera HPLC system (Shimadzu Scientific Instruments, Inc., USA) equipped with a SPD-M40 photodiode array detector on an Agilent Zorbax Eclipse XDB-C18 (4.8 × 150 mm, 5 μm) column. The total separating time was 50 min, and the wavelength was detected at 330 nm. The column temperature was maintained at 30°C with a gas flow of 0.7 ml/min, and the chromatogram was monitored using a detection wavelength of 242 nm. The injection volume was 20 μl. The retention period and UV spectra of the guanosine (100 μg/ml) and uridine (100 μg/ml) standards (Sigma-Aldrich, USA) corresponded with those of the identical peak in the extract.

The liquid chromatography-mass spectrometry (LC-MS) analysis was performed using a Thermo Scientific LTQ-Orbitrap XL LC–MS instrument. This system is composed of a Thermo Scientific Ultimate 3000 UPLC system mass spectrometer. The samples were eluted using a Acquity UPLC C18 (2.1 mm × 150 mm, 1.7 μm, Waters) with the column set to 35°C. All samples were kept refrigerated to 4°C in the UPLC autosampler and a 2 μl injection volume was used with a total flow rate of 0.4 ml/min over a total run time of 30 min. All solvents used were LC–MS grade and ultra-pure water was used for each step. Mobile phase A consisted of water + 0.1% formic acid while mobile phase B was acetonitrile + 0.1% formic acid.

### Cell and Growth Conditions

HaCaT cells (human epidermal keratinocyte) were kindly provided by Prof. Dae Kyun Chung at Kyung Hee University. RAW 264.7 cells (murine macrophages) were purchased from the Korea Cell Line Bank (KCLB, Republic of Korea). HaCaT and RAW 264.7 cells were cultured in DMEM and RPMI-1640 medium. All media comprised 10% fetal bovine serum (FBS) and 1% antibiotic mix of penicillin and streptomycin, and they were cultivated under a humidified 5% CO_2_ atmosphere (MCO-175, Panasonic, Japan) at 37°C.

### Sample Treatment

Prior to treating the sample with cells, the sample was submerged in distilled water (DW) and diluted with a medium supplied with 1% FBS and 1% antibiotic mix at various dosages of CFE (12.5, 25, 50, and 100 μg/ml). RAW 264.7 cells and HaCaT cells were treated with CFE (12.5-100 μg/ml) or aspirin (200 μg/ml) and dexamethasone (DEX, 20 μg/ml). Following 1 h incubation, the treated cells were stimulated with LPS (1 μg/ml) for RAW 264.7 cells and stimulated with TNF-α/IFN-γ (each 10 ng/ml) for HaCaT cells. The sample-treated cells were incubated for 24 h at 37°C.

### Cell Viability Assay

Cell viability was assessed using WST assay with the EZ-cytox Cell Viability Assay Kit (Daeil Lab Service Co., Republic of Korea). RAW 264.7 cells or HaCaT cells at a density of 1 × 10^5^ cells/well in a 96-well plate were exposed to CFE or positive drugs before being subjected to stimulation with LPS or TNF-α/IFN-γ. After 24 h incubation, the culture medium of treated cells was removed and added to 100 μl of EZ-cytox solution for 1 h at 37°C. After that, the absorbance at 450 nm was detected by an EPOCH 2 microplate reader (Agilent BioTek, USA). The rate of cell viability (%) by the setting medium (untreated cells) at 100% was obtained using the following formula:

$$\text{Cell viability (\%) = }\frac{{\text{A}}_{450}\text{ of the sample-tested cells}}{{\text{A}}_{450}\text{ of the untreated cells}}\times 100$$

### Nitric Oxide (NO) Production Assays

The Griess reagent was used to determine the NO content (Promega, USA). RAW 264.7 cells at a density of 1 × 10^5^ cells/well in a 96-well plate were treated with CFE or positive drugs before LPS stimulation for 24 h. The culture supernatant (100 μl) was combined with the Griess reagent following the manufacturer’s instructions. The absorbance at 540 nm was detected and calculated for NO production (%) by setting LPS at 100% as follows:

$$\text{NO production (\%) = }\frac{{\text{A}}_{540}\text{ of the sample-tested cells}}{{\text{A}}_{540}\text{ of the LPS-treated cells}}\times 100$$

### Quantitative Real-Time PCR Analysis

Both cell lines (1 × 10^5^ cells/well) in a 24-well plate were treated with CFE or positive drugs before being induced with LPS and TNF-α/IFN-γ, respectively. After treatment, total RNA was extracted from the cells using TRIzol reagent and then cDNA was synthesized according to the manufacturer’s instructions using the High-Capacity cDNA Reverse Transcription Kit (Applied Biosystems, USA). The mRNA levels of *IL-1β, IL-6, IL-4, TNF-α, iNOS, COX-2*, and *β-actin* in RAW 264.7 cells and those of *IL-4, FLG, IVL, LOR, TSLP*, and *GAPDH* in HaCaT cells were determined using a QuantStudio 3 FlexReal-Time PCR System (Applied Biosystems). A mixture of 5 ng of cDNA, 0.4 μM of each specific primer (Table S1), and TB Green premix Ex Taq TM II (TaKara Bio Inc., Japan) was used for the PCR reaction.

### Western Blot Analysis

RAW 264.7 cells (2 × 10^5^ cells/well) were treated with CFE or positive drugs before LPS stimulation. Western blot analysis was performed by extracting total protein using RIPA lysis buffer (Tech & Innovation, China) with an EDTA solution and protein inhibitor cocktail (Thermo Fisher Scientific, USA). The Pierce BCA Protein Assay Kit (Thermo Fisher Scientific) was used to evaluate the protein content. Protein (30 μg) was separated using 10% SDS-PAGE before being transferred to PVDF membranes. After transfer, the membranes were immobilized with 5%skimmed milk in TBST buffer and placed with primary antibodies against p-NF-κB65, p-p38, p-JNK, p-ERK1/2 (Cell Signaling Technology, USA), and α-tubulin (Abcam, UK) before being immediately followed by incubation with secondary antibodies against goat anti-rabbit IgG(H+L)-HRP (GenDEPOT, USA). The detection of protein bands was performed using Pierce ECL Western Blotting Substrate (Thermo Fisher Scientific), and the signal intensity was determined with the ChemiDoc XRS+ imaging system (Bio-Rad, USA).

### Statistical Analysis

Statistical differences were assessed using IBM SPSS statistics (SPSS, USA). Data were subjected to analysis using one-way analysis of variance, which was subsequently evaluated by Duncan's multiple range test at *p* < 0.05. The results are presented as mean ± standard deviation (SD).

## Results and Discussion

### Identification of Major Compounds in CFE

Guanosine and uridine are naturally produced nucleosides that comprise two nucleobases, uracil and guanine [26]. Both guanosine and uridine are two important components that have been extracted from marine algae [27]. They exhibit anti-inflammatory properties against lung inflammation and asthma in both the in vivo and in vitro systems [28, 29] . In this present study, guanosine and uridine were used as volatile markers for the phytochemical study of CFE on the LC-MS system. The major compounds of CFE, which contain guanosine and uridine, are presented in Fig. S2. As shown in Fig. S2A and S2B, a representative LC-MS chromatogram of the standards of guanosine (100 μg/ml) and uridine (100 μg/ml) at 242 nm were observed. CFE showed the retention times of guanosine at 5.118 ± 0.022 min and uridine at 4.621 ± 0.012 min, which matched with the standard (Fig. S2C).

### CFE Activates Cell Viability and NO Production in RAW 264.7 Cells

**Fig. 1:**
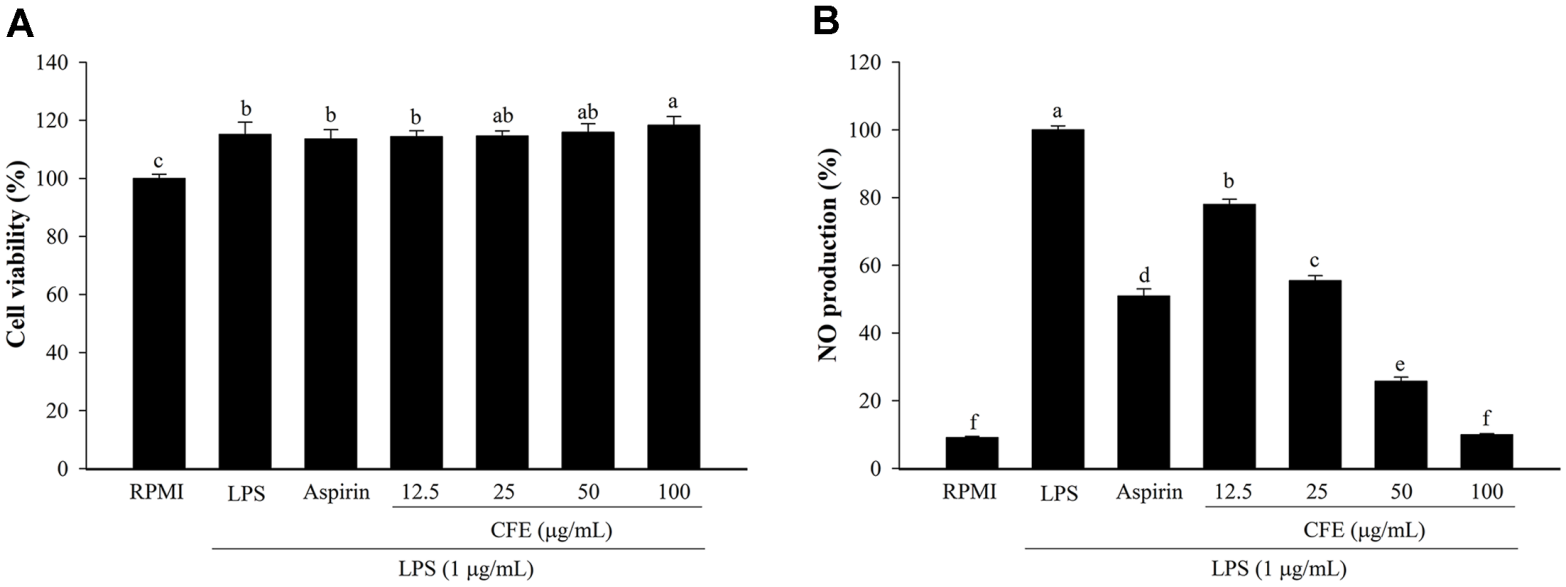
Effects of CFE on cell cytotoxicity (**A**) and NO production (**B**) in LPS-stimulated RAW264.7 macrophages. Following pretreatment of cells with CFE (12.5–100 μg/ml) or aspirin (200 μg/ml) and then exposure to LPS (1 μg/ml). The RPMI (untreated cells) value was obtained in the absence of LPS and CFE. Values are presented as mean ± SD (*n* = 3). The letters ^a-f^ indicate significant differences (*p* < 0.05) between treatment groups.

The cellular viability assay was the preliminary investigation to explore the cytotoxic effect of CFE on RAW 264.7 cells. CFE showed no cytotoxicity at any concentration compared with that in the RPMI-treated cells (Fig. 1A). Similarly, positive controls, such as LPS, and positive drugs, such as aspirin, had no cytotoxic effect and their doses were higher than that of RPMI (*p* < 0.05). As a result, CFE doses ranging from 12.5 to 100 μg/ml were chosen for studying the anti-inflammatory effects.

NO is an endogenous free radical that plays a significant signaling role in a range of physiological and pathological functions [30]. As shown in Fig. 1B, CFE significantly decreased NO generation induced by LPS by 77.99%, 55.45%, 25.74%, and 9.95% at doses of 12.5, 25, 50, and 100 μg/ml, respectively. Compared with RPMI, CFE at a dose of 100 μg/ml showed a strong inhibitory effect on LPS-stimulated NO production, similar to that produced by RPMI (9.08%). Aspirin acted as a positive drug and displayed significant NO activity inhibition by 50.93%. Aspirin, also known as acetylsalicylic acid, is a common drug for treating pain, fever and inflammation and is also frequently studied for its anti-inflammatory effect on LPS-induced macrophages [31-33]. The current study found that CFE could significantly alleviate inflammation by reducing these inflammatory mediators in LPS-induced macrophages.

### CFE Inhibits the Expression of Inflammatory Mediators and Cytokines in RAW 264.7 Cells

**Fig. 2:**
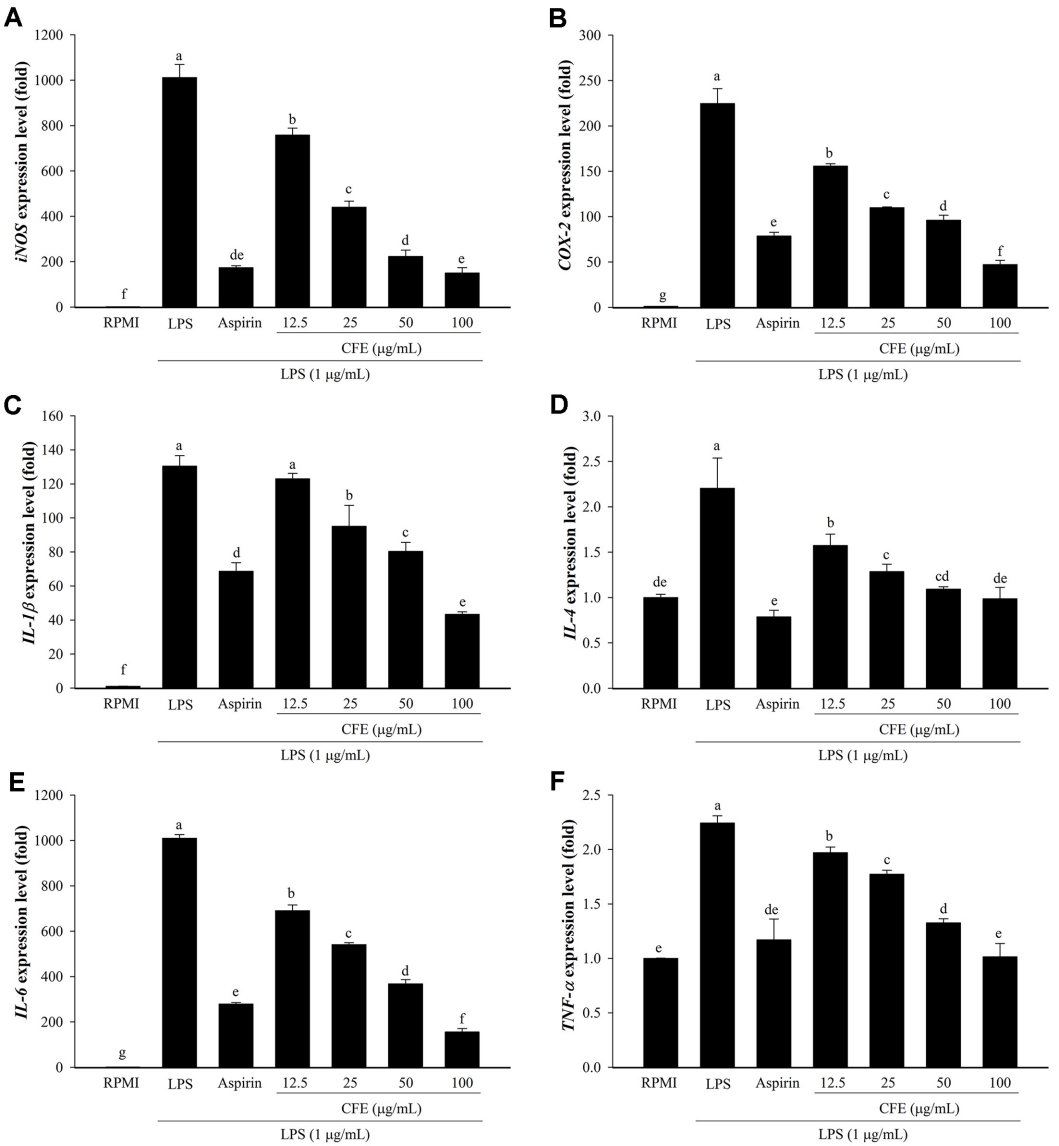
Effects of CFE on mRNA expression levels of inflammation-associated genes in RAW264.7 cells. The mRNA expression levels of *iNOS* (**A**), *COX-2* (**B**), IL-1β (**C**), *IL-4* (**D**), *IL-6* (**E**), and *TNF-α* (**F**) were determined by real-time PCR. Following pretreatment of cells with CFE (12.5–100 μg/ml) or aspirin (200 μg/ml) and then exposure to LPS (1 μg/ml). The RPMI (untreated cells) value was obtained in the absence of LPS and CFE. Values are presented as mean ± SD (*n* = 3). The letters ^a-g^ indicate significant differences (*p* < 0.05) between treatment groups.

In order to evaluate the anti-inflammatory effect of CFE, real-time PCR analysis was used, and it showed that CFE reduced the mRNA expression levels of *iNOS, COX-2, IL-1β, IL-4, IL-6*, and *TNF-α*. Macrophages play a critical role in inflammatory disorders caused by the overproduction of NO and PGE_2_ by activating iNOS and COX-2 [13, 32]. As shown in Fig. 2A and 2B, LPS considerably increased the mRNA expression levels of the inflammatory mediators *iNOS* and *COX-2* compared with RPMI. In comparison to LPS alone, the mRNA levels of *iNOS* and *COX-2* were dramatically downregulated with the addition of CFE, and the effects of CFE were the most noticeable at a dose of 100 μg/ml. Several studies suggest that the plant extract may inhibit cytokines involved in inflammation and can also be used as a skin inflammation treatment [34-36]. Therefore, these findings imply that CFE may suppress inflammation-regulated genes, providing a possible therapy for treating skin inflammation.

In response to LPS, macrophages secrete TNF-α, which promotes the release of other pro-inflammatory cytokines, such as IFN-γ, IL-1β, IL-2, IL-4, IL-6, and IL-10 [37]. *Chrysanthemum indicum* extract has been shown to inhibit the expression of *TNF-α, IL-1β, IL-6, iNOS*, and *COX-2* in LPS-induced macrophages [13]. Treatment with CFE (12.5–100 μg/ml) significantly suppressed LPS-induced expression of *IL-1β, IL-4, IL-6*, and *TNF-α* (Fig. 2C-2F). In addition, all six inflammatory genes were dramatically downregulated by CFE when compared with LPS and the results showed that CFE led to stronger inhibition effects than aspirin. Natural compounds have shown a stronger inhibitory effect than LPS and aspirin on the expression of these genes in many studies [31, 32]. Consistent with previous reports, the current findings imply that CFE reduces the synthesis of inflammatory factors and pro-inflammatory cytokines at the transcriptional level.

### CFE Inhibits MAPK and NF-κB Activation in RAW 264.7 Cells

**Fig. 3:**
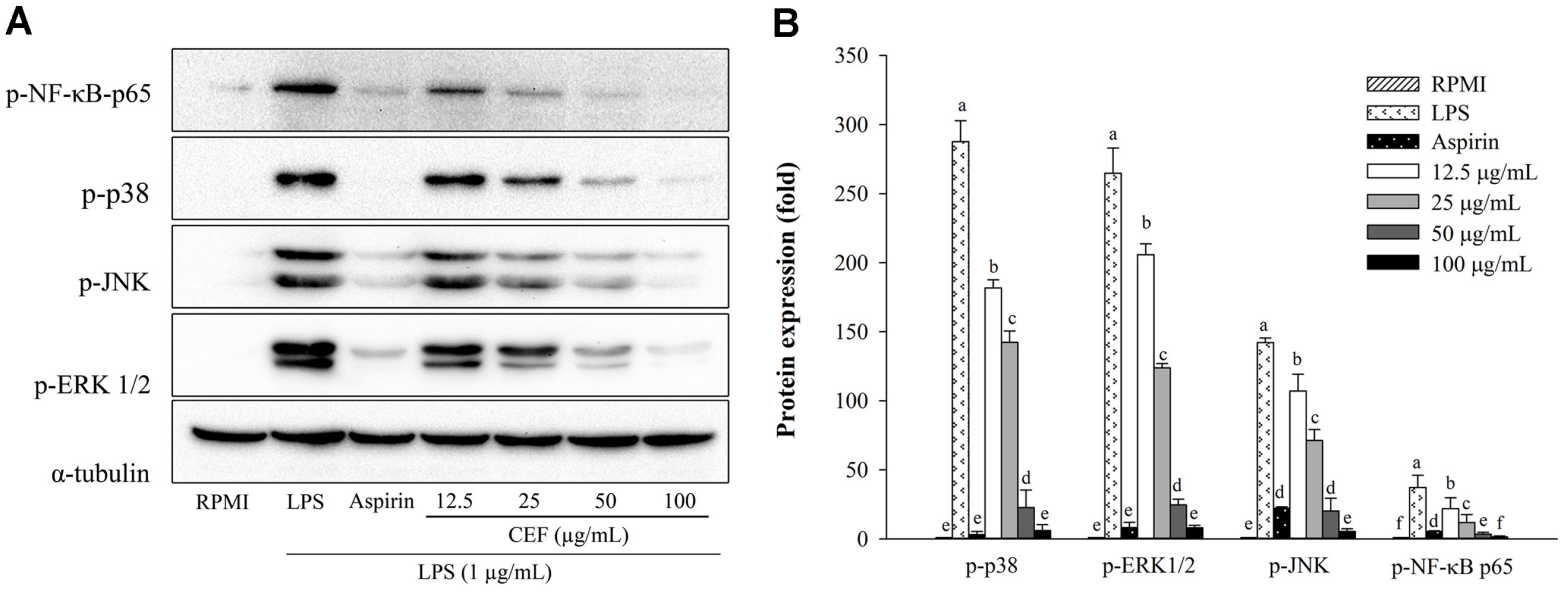
Effects of CFE on the phosphorylation of NF-κB and MAPK in RAW264.7 cells. Western blot analysis was used to identify the phosphorylated proteins that were expressed on the blot (**A**). The relative protein levels of the NF-κB and MAPK pathways (**B**). Following pretreatment of cells with CFE (12.5–100 μg/ml) or aspirin (200 μg/ml) and then exposure to LPS (1 μg/ml). The RPMI (untreated cells) values were obtained in the absence of LPS and CFE. Values are presented as mean ± SD (*n* = 3). The letters ^a-f^ indicate significant differences (*p* < 0.05) between treatment groups.

The MAPK signaling molecules include p38, ERK, and JNK, which are essential for cell division and proliferation, and the transcription factor NF-κB promotes the expression of inflammatory cytokines and mediators, such as COX-2 and iNOS, which play a vital role in the immune system [38, 39]. Macrophages respond to LPS stimulation by phosphorylating and subsequently degrading IκB, which triggers NF-κB activation and nuclear translocation [38]. Fig. 3 shows that the protein expression level of the NF-κB subunit p65 was significantly elevated following LPS treatment alone, indicating that LPS triggered NF-κB-p65 translocation from the cytosol to nucleus. On the other hand, CFE reduced the production of LPS-induced phosphorylated NF-κB-p65 depending on the concentration (Fig. 3). Furthermore, LPS stimulation promoted phosphorylation of ERK1/ 2, JNK, and p-38, whereas CFE reduced LPS-induced phosphorylation of these kinases in a dose-dependent manner (Fig. 3). These results revealed that CFE effectively inhibited the phosphorylation of the two pathways in RAW 264.7 macrophages, resulting in its anti-inflammatory effects.

### CFE Activates Cell Viability in HaCaT Cells Stimulated by TNF-α/IFN-γ

**Fig. 4:**
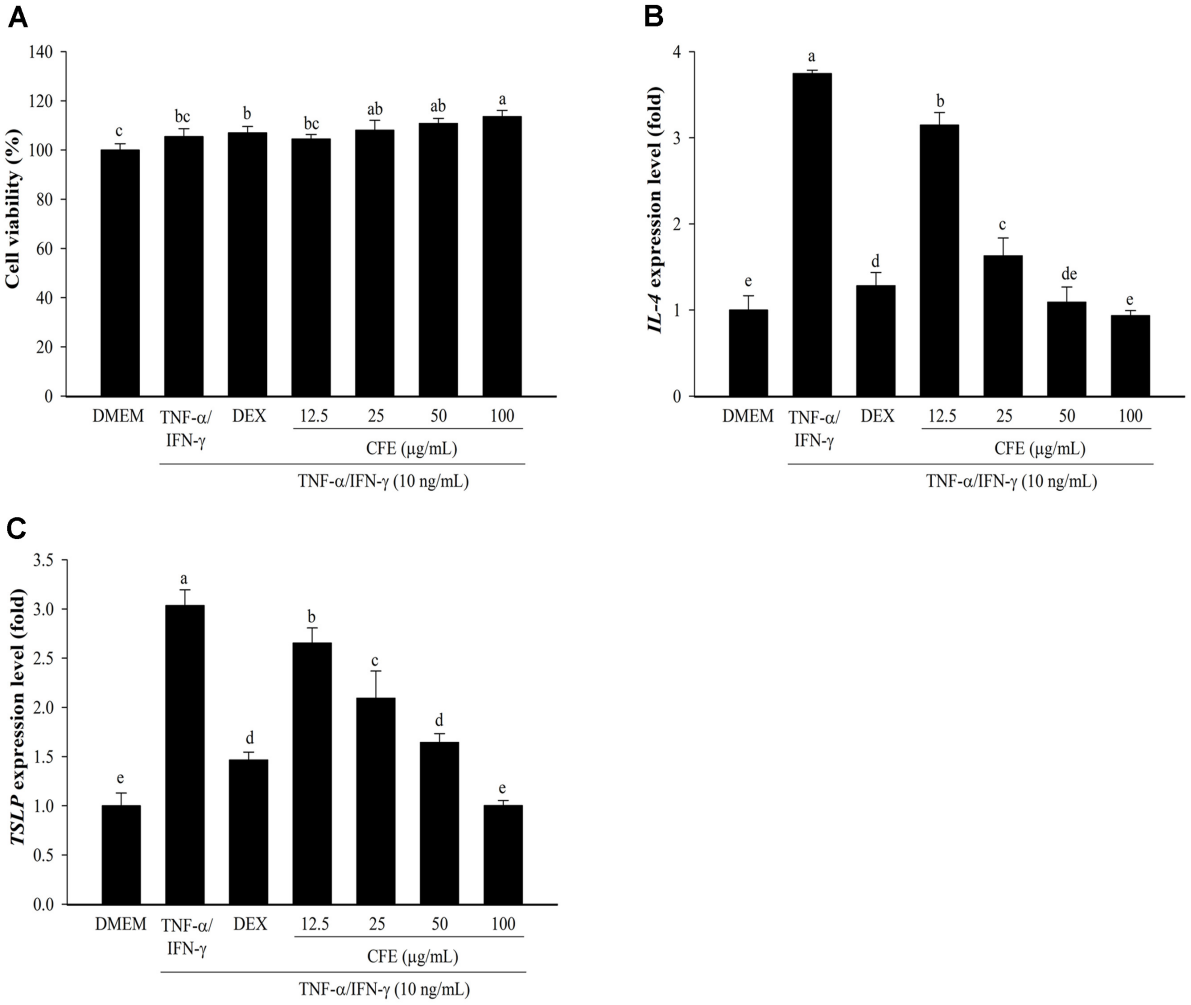
Effects of CFE on cell viability and skin inflammation expression in HaCaT cells. The cytotoxic ability in HaCaT cells (**A**) was evaluated by the WST assay. The mRNA expression levels of *IL-4* (**B**) and *TSLP* (**C**) were determined by real-time PCR. Following pretreatment of cells with CFE (12.5–100 μg/ml) or Dex (20 μg/ml) and then exposure to TNF-α/ IFN-γ (10 ng/ml). DMEM (untreated cells) values were obtained in the absence of TNF-α/IFN-γ and CFE. Dex, Dexamethasone. Values are presented as mean ± SD (*n* = 3). The letters ^a-e^ indicate significant differences (*p* < 0.05) between treatment groups.

Keratinocytes, the major component of epidermal cells, are responsible for forming a skin barrier through keratinization and differentiation [4]. Keratinocytes activated by a variety of triggers serve an essential role in immune responses [40]. To evaluate the impact of CFE on skin inflammation, the cellular cytotoxicity of HaCaT cells was evaluated by treatment with CFE and stimulation with TNF-α/IFN-γ. The results showed that CFE did not affect the viability of HaCaT cells at all concentrations (Fig. 4A). CFE activated cell viability by 104.52–113.62%, depending on the concentration (12.5–100 μg/ml), which was higher than that in the untreated cells (DMEM). DEX was used as a positive drug, and it showed cellular toxicity of 107.03%. DEX has been used to treat multiple sclerosis, allergies, cerebral edema, inflammation, and shock [41]. Many studies have used DEX as a positive drug to evaluate its anti-inflammatory effects and atopic dermatitis [42, 43]. Therefore, CFE at a concentration range of 12.5–100 μg/ml was used for subsequent experiments to evaluate skin inflammation in TNF-α/IFN-γ- stimulated HaCaT cells.

### CFE Inhibits the Expression of Cytokines Related to Skin Inflammation in HaCaT Cells

Further studies were conducted with nontoxic concentrations of CFE to assess its effects on skin inflammation and barrier function. Previous research has proven that cytokines, such as IL-4 and IL-13, play significant roles in the pathophysiology of AD, and they are the key therapeutic targets [44]. TSLP is a cytokine that contributes to skin barrier defects and modulates inflammation in food allergies, asthma, and AD. It is extensively produced by keratinocytes in skin lesions in both acute and chronic AD patients [45, 46]. It was also observed that *Schisandra chinensis* extract increased the levels of TNF-α, IL-1β, IL-4, IL-6, IL-8, monocyte chemotactic protein (MCP)-1, and TSLP in mice [47].

As shown in Fig. 4B and 4C, CFE effectively inhibited the *IL-4* and *TSLP* expression in a dose-dependent manner in HaCaT cells stimulated by TNF-α/IFN-γ. The stimulation by TNF-α/IFN-γ in HaCaT cells alone upregulated the mRNA levels of both genes when compared with DMEM. Dexamethasone also showed 1.28 ± 0.15 and 1.47 ± 0.08-fold *IL-4* and *TSLP* expression, respectively. In addition, CFE at a dose of 100 μg/ml showed a significant inhibitory effect, similar to DMEM. Consequently, these findings suggest that CFE markedly reduced the expression of *IL-4* and *TSLP*, which were produced by TNF-α/IFN-γ.

### CFE Promotes the mRNA Expression of Factors Related to Skin Barrier Function in HaCaT Cells

It is well known that FLG, LOR, and IVL are important factors in the structure and function of the skin barrier [6]. The reduction of IVL, LOR, and FLG is the primary characteristic of AD lesional skin, which is related to skin barrier dysfunction [48]. Furthermore, they are important proteins for normal epidermal homeostasis, and their expression control can be used to assess the efficacy of enhancing the skin barrier function [49]. Normally expressed in keratinocytes, FLG plays a role in epidermal differentiation and skin barrier function, which have been implicated in AD [50, 51]. IVL and FLG showed effect on skin hydration and barrier function and moderate UV protection in HaCaT cells [52].

**Fig. 5:**
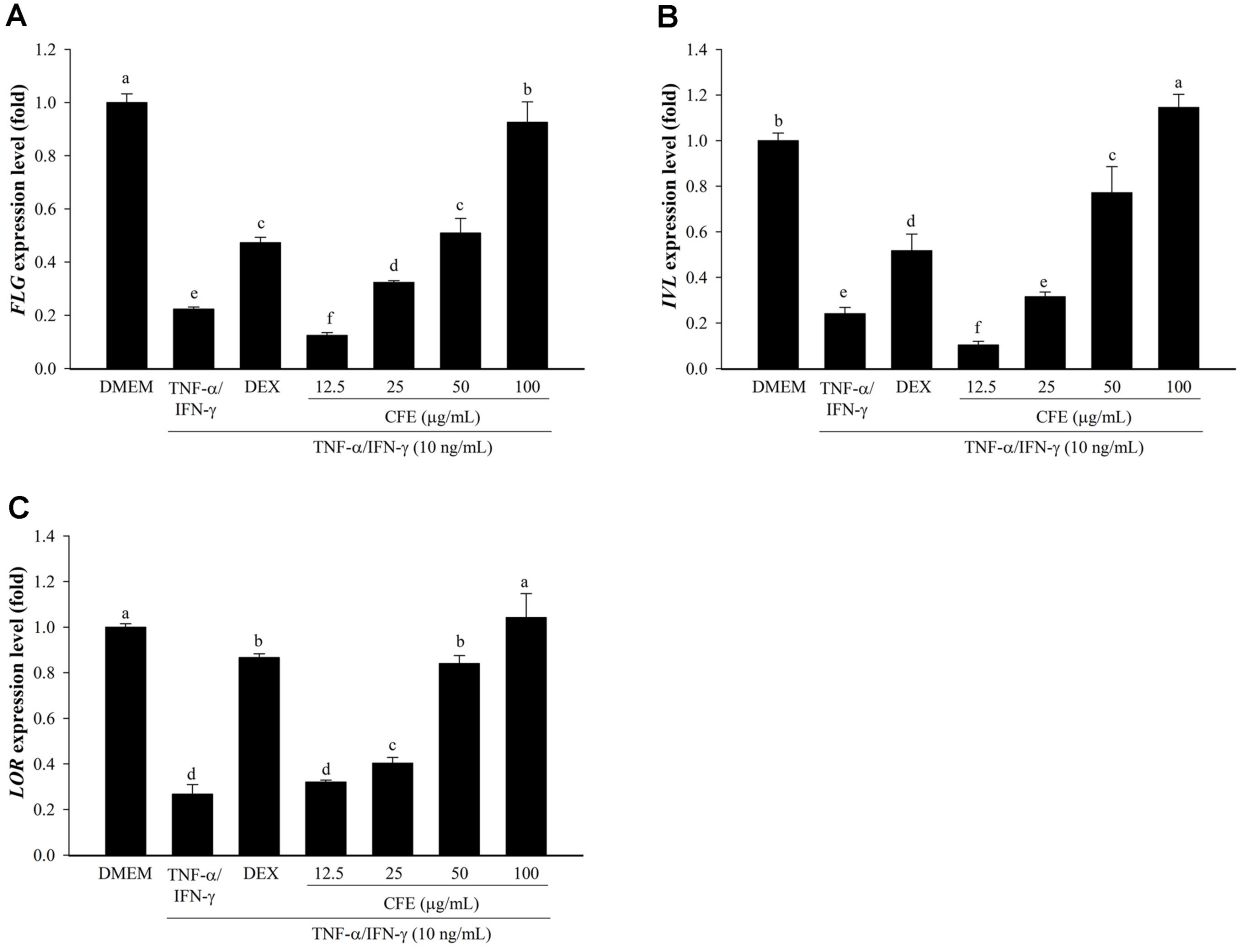
Effects of CFE on the expression of skin barrier factor in HaCaT cells. The mRNA expression levels of *FLG* (**A**), *IVL* (**B**), and *LOR* (**C**) were determined by real-time PCR Following pretreatment of cells with CFE (12.5–100 μg/ml) or Dex (20 μg/ml) and then exposure to TNF-α/IFN-γ (10 ng/ml). DMEM (untreated cells) values were obtained in the absence of TNF-α/IFN-γ and CFE. Dex, Dexamethasone. Values are presented as mean ± SD (*n* = 3). The letters ^a-f^ indicate significant differences (*p* < 0.05) between treatment groups.

In this study, real-time PCR was used to measure the expression of factors related to skin barrier function. Fig. 5 shows that the *FLG*, *LOR*, and *IVL* expression levels were increased by CFE in TNF-α/IFN-γ stimulated HaCaT cells. Compared to DMEM, the mRNA expression of these three factors was improved by CFE, which is consistent with previous research [43, 53]. In particular, high recovery was shown at a dose of 100 μg/ml. Consequently, these findings revealed that CFE may be a useful medicinal agent for treating chronic inflammation and restoring the biological skin barrier in AD.

## Conclusion

In the present study, our findings indicate that the principal component chemicals, guanosine and uridine, were found in CFE, which had a strong anti-inflammatory effect. CFE showed down-regulation of inflammatory mediators and cytokines by inhibiting NF-κB and MAPK activation in LPS-stimulated RAW 264.7 cells. CFE inhibited *IL-4* and *TSLP* expression in HaCaT cells, skin keratinocytes stimulated with TNF-α/IFN-γ. In addition, it also increased the expression of atopic-related skin barrier factors. Therefore, CFE could be a possible therapeutic ingredient for AD therapy with anti-inflammation and skin barrier-strengthening properties derived from marine natural products. Further investigation must be conducted to assess whether CFE exhibits inflammatory in animal models.

## Supplemental Materials

Supplementary data for this paper are available on-line only at http://jmb.or.kr.
