# Supplementary material for: In Vitro Anti-Inflammatory and Skin Protective Effects of Codium fragile Extract on Macrophages and Human Keratinocytes in Atopic Dermatitis
Source: J Microbiol Biotechnol. 2024 Jan 17;34(4):940–8. doi: 10.4014/jmb.2312.12002 (PMC11091671; doi:10.4014/jmb.2312.12002)
Supplement: Supplementary file 1 [file jmb-34-4-940-supple.pdf]

Figure S1

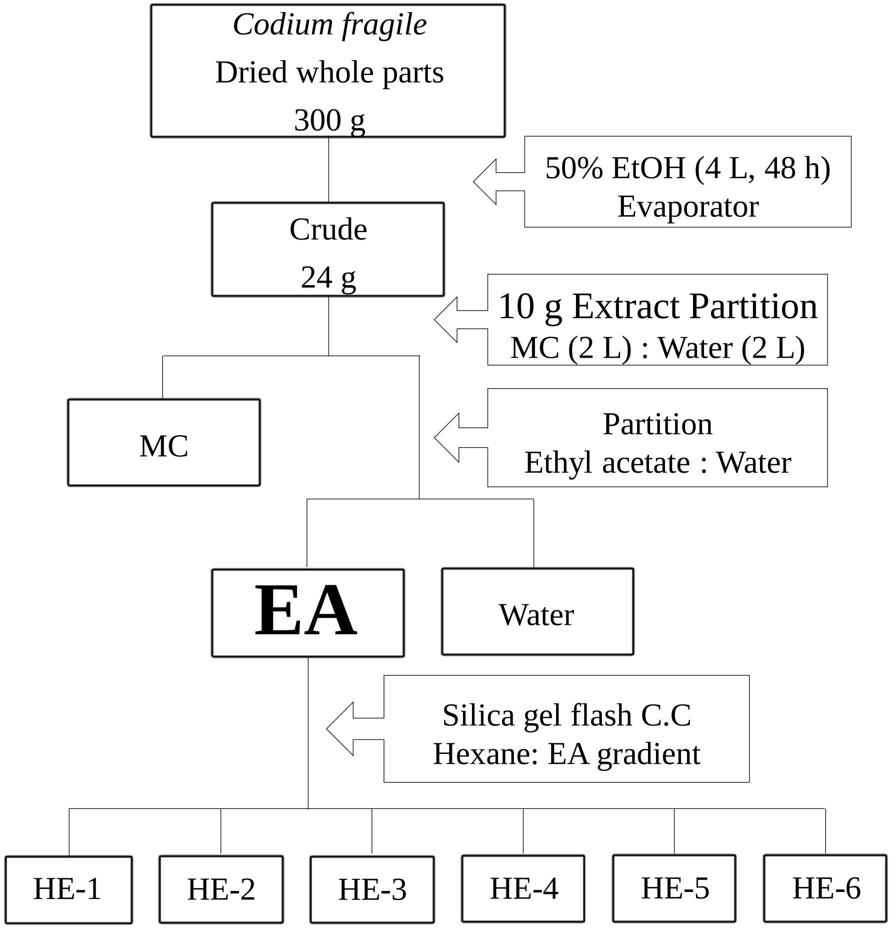

Figure S1. Schematic diagram for extraction process of *C. fragile* extract

MC, Methylene Chloride; EA , Ethyl acetate; HE, the products of EA after separation with silica gel by hexane: EA at different ratios

Figure S2

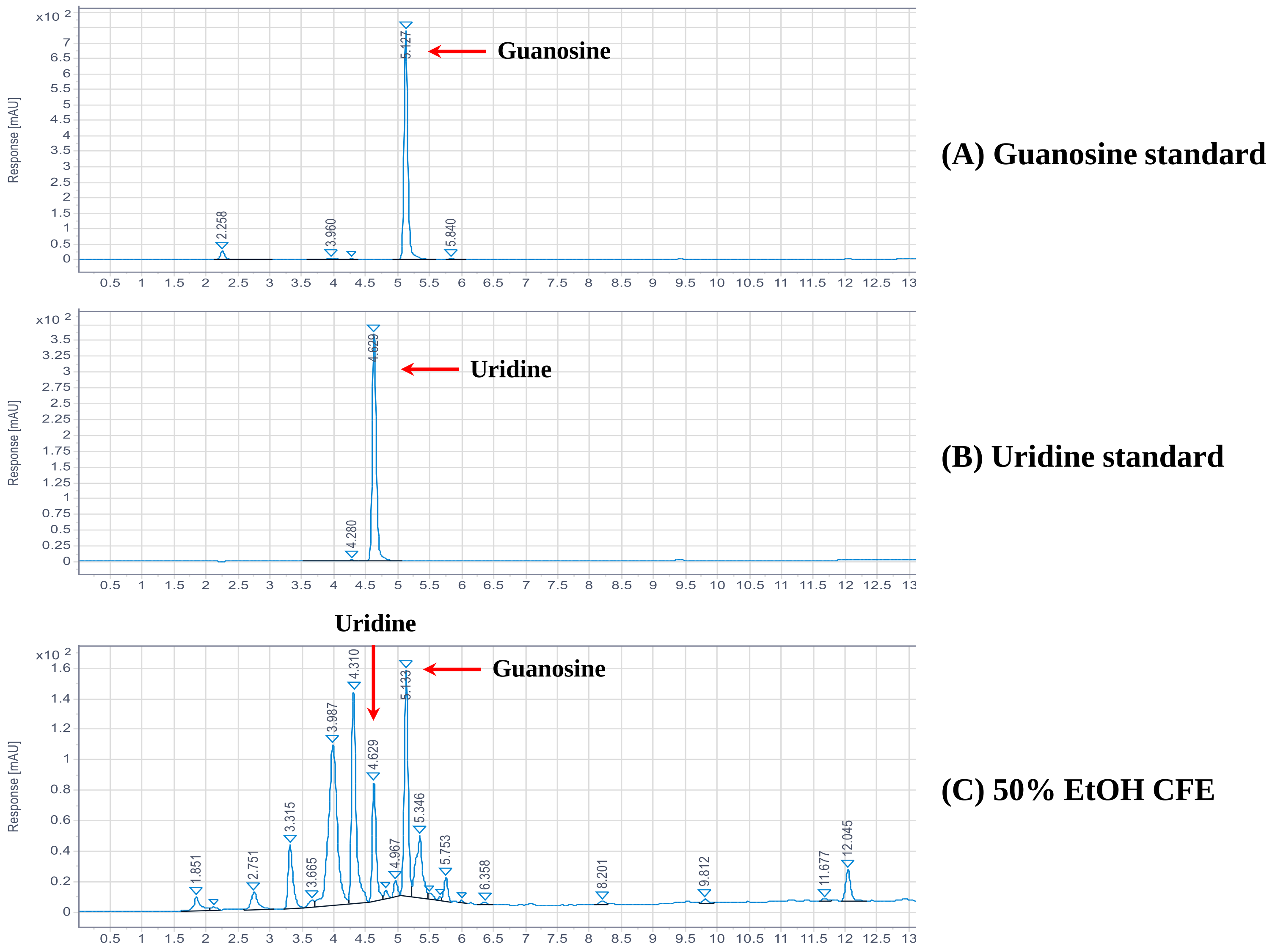

Figure S2. Liquid chromatography mass spectrometry (LC-MS) analysis of *Codium fragile* extract (CFE).

(A) LC-MS chromatograms of guanosine standard. (B) LC-MS chromatograms of uridine standard. (C) LC-MS chromatograms of CFE.

Table S1. Nucleotide primers used in this study

Gene	Primer sequences	Accession number
mIL-1β	F: GGGCCTCAAAGGAAAGAATC<br>R: TACCAGTTGGGGAACTCTGC	NM_008361.4
mIL-6	F: AGTTGCCTTCTTGGGACTGA<br>R: CAGAATTGCCATTGCACAAC	NM_031168.2
mIL-4	F: ACAGGAGAAGGGACGCCAT<br>R: GAAGCCCTACAGACGAGCTCA	NM_021283.2
mTNF-α	F: ATGAGCACAGAAAGCATGATC<br>R: TACAGGCTTGTCACTCGAATT	D84199.2
miNOS	F: TTCCAGAATCCCTGGACAAG<br>R: TGGTCAAACTCTTGGGGTTC	BC062378.1
mCOX-2	F: AGAAGGAAATGGCTGCAGAA<br>R: GCTCGGCTTCCAGTATTGAG	NM_011198.4
mβ-actin	F: CCACAGCTGAGAGGAAATC<br>R: AAGGAAGGCTGGAAAAGAGC	NM_007393.5
hIL-4	F: CCCCTCTGTTCTTCCTGCT<br>R: TCGTCTTTAGCCTTTCCAAG	BC070123.1
hFLG	F: CAGTCAGACTCTAGTACCGCTAAGG<br>R: CACTACCATAGCTGCCATGTCTC	NM_002016.2
hLOR	F: GTGGGAGCGTCAAGTACTCC<br>R: GAGACGCCTCCGTAGCTCTG	M61120.1
hIVL	F: CCCATCAAAGCAAGAGGAAA<br>R: AGCTGCTGATCCCTTTGTGT	NM_005547.4
hTSLP	F: AACCTTCAATCCCACCGCC<br>R: AGGATTGTGGCGTCGCTTC	AY037115.1
hGAPDH	F: ATCAAGAAGGTGGTGAAGCAGGC<br>R: TCAAAGGTG GAGGAGTGGGTGTC	J04038.1

Note: m = mouse, h = human
